# Enhancing osteogenic potential of hDPSCs by resveratrol through reducing oxidative stress via the Sirt1/Nrf2 pathway

**DOI:** 10.1080/13880209.2022.2037664

**Published:** 2022-02-21

**Authors:** Jingying Zhang, Rui Li, Kenny Man, Xuebin B. Yang

**Affiliations:** a Key Laboratory of 3D Printing Technology in Stomatology, The First Dongguan Affiliated Hospital, Guangdong Medical University, Dongguan, China; b College of Physics, Dalian University of Technology, Dalian, China; c School of Chemical Engineering, University of Birmingham, Birmingham, UK; d Biomaterials & Tissue Engineering Group, School of Dentistry, University of Leeds, Leeds, UK

**Keywords:** Dental pulp stromal cell, ROS, SOD, RUNX1, OCN, xCT, skull defect

## Abstract

**Context:**

The osteogenic potential of the human dental pulp stromal cells (hDPSCs) was reduced in the state of oxidative stress. Resveratrol (RSV) possesses numerous biological properties, including osteogenic potential, growth-promoting and antioxidant activities.

**Objective:**

This study investigates the osteogenic potential of RSV by activating the Sirt1/Nrf2 pathway on oxidatively stressed hDPSCs and old mice.

**Materials and methods:**

The hDPSCs were subjected to reactive oxygen species (ROS) fluorescence staining, cell proliferation assay, ROS activity assay, superoxide dismutase (SOD) enzyme activity, the glutathione (GSH) concentration assay, alkaline phosphatase staining, real-time polymerase chain reaction (RT-PCR) and Sirt1 immunofluorescence labelling to assess the antioxidant stress and osteogenic ability of RSV. Forty female Kunming mice were divided into Old, Old-RSV, Young and Young-RSV groups to assess the repair of calvarial defects of 0.2 mL RSV of 20 mg/kg/d for seven days by injecting intraperitoneally at 4 weeks after surgery using micro-computed tomography, nonlinear optical microscope and immunohistochemical analysis.

**Results:**

RSV abates oxidative stress by alleviating the proliferation, mitigating the ROS activity, increasing the SOD enzyme activity and ameliorating the GSH concentration (RSV IC_50_ in hDPSCs is 67.65 ± 9.86). The antioxidative stress and osteogenic capabilities of RSV were confirmed by the up-regulated gene expression of SOD1, xCT, RUNX2 and OCN, as well as Sirt1/Nrf2. The collagen, bone matrix formation and Sirt1 expression, are significantly increased after RSV treatment in mice.

**Discussion and conclusions:**

For elderly or patients with oxidative stress physiological states such as hypertension, heart disease, diabetes, etc., RSV may potentially improve bone augmentation surgery in regenerative medicine.

## Introduction

Cell therapy has gained significant attention as a novel therapeutic approach to restore/repair tissue/organ functionality after injury (Marvasti et al. 2019). Cellular component represents the key factor in cell-based therapy, particularly in using mesenchymal stromal cells (MSCs), which is considered the gold standard for clinical research (Berebichez-Fridman and Montero-Olvera 2018). Although MSCs can hypothetically be obtained from different and accessible adult tissues, there are practical limitations concerning the invasive procedure, low procurement yield, donor site morbidity, extensive *in vitro* expansion and various donor characteristics (Berebichez-Fridman and Montero-Olvera 2018). A number of studies showed that the human dental pulp stromal cells (hDPSCs) are easily accessible via discarded medical waste (Gronthos et al. 2000) and have greater proliferative and osteogenic potential than the MSCs derived from bone marrow, making them favourable as an alternative MSC source for cell-based therapies (El-Gendy et al. 2013; Jensen et al. 2016).

Recent evidence indicated that stem cells might undergo progressive senescence or lose their regenerative capacity and experience gradual exhaustion of their characteristic proliferative function. Oxidative stress refers to a functional inability of the reactive oxygen species (ROS) in performing the detoxification of metabolic reactive intermediates of biological systems (Forrester et al. 2018). Three important categories of ROS, hydrogen peroxide (H_2_O_2_), hydroxyl radicals (•OH) and superoxide anion (O^2−^), generated through different signalling pathways are reported to be beneficial for cellular processes such as cellular proliferation and differentiation (Guan et al. 2019; Li et al. 2019). Several studies have demonstrated that persistent intracellular accumulation of H_2_O_2_ and exogenous exposure to H_2_O_2_ could induce cellular senescence (Song et al. 2014; Zhu et al. 2015; Gao et al. 2017). Therefore, it is necessary to explore the osteogenic potential of hDPSCs in oxidative stress.

Resveratrol (RSV; *trans*-3,5,4′-trihydroxystilbene), a naturally occurring nonflavonoid polyphenolic compound, richly presents in many fruits and vegetables, such as peanuts, mulberries and grapes (Gambini et al. 2013; Rauf et al. 2017). It has been reported that RSV can control the differentiation of lineage-specific neural stem cells and mediate the biological functions of terminally differentiated cells (Hu et al. 2014). Besides, RSV is also known as the Sirt1 activator (Wood et al. 2004) and has attained wide usage in dietary supplementation and traditional medicine (Biswas et al. 2020; Wu et al. 2020). Studies have shown that RSV exerts substantial antioxidant effects through scavenging excessive free radicals and enhancing the biosynthesis of intracellular antioxidant enzymes (Shakibaei et al. 2012; Kulkarni and Canto 2015; Sadi et al. 2018). Besides, RSV may prevent metabolic diseases through the activation of Sirt1, which further deacetylate the mitochondrial co-enzyme PGC-1α and improve mitochondrial functions (Lagouge et al. 2006). The mechanism of RSV regulating osteogenic potential in hDPSCs in oxidative stress needs further exploration. Therefore, we hypothesize that RSV could promote the osteogenesis of hDPSCs in oxidative stress via activating the Sirt1–Nrf2 pathway.

## Materials and methods

### Cell culture

A total of eight teeth from different donors obtained with patients’ informed consent according to the current study, which had approval from the Research Ethics Committee (YS2020147) were collected separately, and the hDPSCs were extracted according to the method as described in our previous article (Zhang et al. 2019). The hDPSCs were cultured with growth medium (α-modified minimum essential medium (α-MEM) supplemented with 10% fetal bovine serum (FBS), 1% penicillin/streptomycin and 2 mM l-glutamine). All reagents were obtained from Gibco (Carlsbad, CA), and the cells were cultured in an incubator at 37 °C with 5% CO_2_ (Binder, Tuttlingen, Germany). The medium was changed every three days, and the cells were treated with trypsin when they reached 80% confluence. The hDPSCs of passages 3–5 were used for the following experiments.

### Oxidative stress cell model and regents

The oxidative stress model was optimized by different concentrations of H_2_O_2_ (0.1, 0.2 and 0.3 mM) treated for 24 h. All H_2_O_2_ solutions were prepared freshly. The RSV (Sigma-Aldrich, Shanghai, China) was dissolved in dimethyl sulfoxide (10 µL) (DMSO, Sigma-Aldrich, Shanghai, China) and diluted to 10 μM final concentration in a growth medium. The hDPSCs (*n* = 8) were divided into four groups. Normal control (NC) group refers to the hDPSCs which were cultured with growth medium, RSV group refers to the hDPSCs which were treated with RSV, H_2_O_2_ group were the hDPSCs which were cultured with H_2_O_2_ (0.2 mM), and H-RSV refers to the hDPSCs which were cultured with 0.2 mM H_2_O_2_ and 10 μM RSV.

### Intracellular ROS fluorescent staining

The cells at 2.5 × 10^4^ cells/cm^2^ (*n* = 6) were seeded and attached to eight-chamber glass coverslips (Corning, Falcon culture slides, Corning, NY). Peroxide-sensitive dye 2′,7′-dichlorodihydrofluorescein diacetate (H_2_DCF-DA) (50 μg) (Invitrogen™, Carlsbad, CA) was dissolved in DMSO (10 μL) and were diluted with the serum-free medium at a final concentration of 17.4 μM. The cells were incubated at 37 °C in darkness for 30 min and washed with 1 × PBS three times. The images were acquired by inverted fluorescence microscopy (ZEISS, AX-10, Oberkochen, Germany).

### Cell proliferation assay

Cell proliferation assay was determined by MTT Cell Proliferation and Cytotoxicity Assay Kit (Solarbio, Beijing, China). Briefly, the cells were seeded in 96-well plates at 2.5 × 10^4^ cells/cm^2^ (*n* = 6) and cultured in a growth medium for 24 h. MTT reagent (10 μL) and serum-free basal media (90 μL) were added to each well and incubated for 4 h. The supernatant was aspirated; formazan solutions (110 μL) were added to each well to dissolve the MTT formazan. Absorbance was measured at 490 nm using the Spectra Max^®^ Plus Absorbance Microplate Reader (Molecular Devices, Sunnyvale, CA).

### ROS activity

The intracellular ROS activity was examined by the Reactive Oxygen Species Assay Kit (Beyotime Biotechnology, Shanghai, China). Briefly, the cells were seeded at the density of 2.5 × 10^4^ cells/cm^2^ in 96-well plates and cultured for 24 h (*n* = 3). DCFH-DA was diluted 1:1000 in serum-free medium to 10 μmol/L concentration and added to each well. The cells were incubated at 37 °C for 20 min and washed three times with a serum-free medium. The ROS activity was measured at 488 nm by Spectra Max^®^ Plus Absorbance Microplate Reader (Molecular Devices, Sunnyvale, CA).

### SOD enzyme activity

A total superoxide dismutase (SOD) assay kit with water-soluble tetrazolium salt, WST-1 (Beyotime Biotechnology, Shanghai, China), was used to analyse the SOD enzyme activity following the manufacturer's instructions. Briefly, the cells were seeded at a density of 2.5 × 10^4^ cells/cm^2^ in 96-well plates (*n* = 3) and treated as described previously. The cells were harvested, lysed in 1 × PBS by ultrasonic pyrolysis, and centrifuged at 2200 rpm for 5 min at 4 °C. The supernatant (100 μL) was mixed with SOD working solution (160 μL) at 4 °C. The reaction mixture was centrifuged and transferred to 96-well plates, and the absorbance values were measured at 450 nm using a Spectra Max^®^ Plus Absorbance Microplate Reader (Molecular Devices, Sunnyvale, CA).

### Glutathione (GSH) concentration assay

A total glutathione assay kit (Nanjing Jiancheng Bioengineering Institute, Nanjing, China) with enzyme labelling was used to analyse GSH following the manufacturer's instructions. Briefly, the cells were seeded at 2.5 × 10^4^ cells/cm^2^ in 24-well plates (*n* = 3) and treated as described previously. The cells were harvested, lysed in 1 × PBS by ultrasonic pyrolysis. The supernatant (100 μL) was mixed with precipitant agents (100 μL) and centrifuged at 3500 rpm for 10 min at 25 °C. Following this, the reaction mixture was kept at room temperature for 5 min, and the absorbance values were measured at 405 nm using a Spectra Max^®^ Plus Absorbance Microplate Reader (Molecular Devices, Sunnyvale, CA).

### Quantitative real-time PCR (qRT-PCR)

The total RNA was extracted from the cells using RNAiso Plus reagent (TaKaRa, Kusatsu, Japan). RNAiso Plus (1 mL) was added to each culture dish (6 cm diameter) (*n* = 4) to lyse the cells. Total RNA (1 µg) was reverse transcribed using a PrimeScript TM RT reagent Kit with gDNA Eraser Kit (TaKaRa, Kusatsu, Japan). To quantify the mRNA expression, an amount of cDNA equivalent to the total RNA reacting system (20 µL) was amplified using SYBR Premix Ex Taq TM (TliRNase H Plus, TaKaRa, Kusatsu, Japan) following the manufacturer's protocol. The transcript levels of genes were evaluated with β-actin serving as the housekeeping internal control. The primer sequences are listed in Table 1. Relative transcript levels were calculated as 2^–ΔΔCt,^ in which ΔΔCt = Δ*E* – Δ*C*, Δ*E*=Ct_exp_ – Ct_β-actin_ and Δ*C*=Ct_ct1_ – Ct_β-actin_.

**Table 1.** Primer of targeting genes.

Gene		Primer
β-actin	Forward	5′-AGTTGCGTTACACCCTTTC-3′
	Reverse	5′-TGTCACCTTCACCGTTCC-3′
SOD1	Forward	5′-AGTGCAGGGCATCATCAATTTC-3′
	Reverse	5′-CCATGCAGGCCTTCAGTCAG-3′
xCT	Forward	5′-GGCTATGTGCTGACAAATGTGG-3′
	Reverse	5′-GGGCAACAAAGATCGGAACTG-3′
Runx2	Forward	5′-GAGGCAAGAGTTTCACC-3′
	Reverse	5′-CAGAGTTCAGGGAGGG-3′
OCN	Forward	5′-GGCAGCGAGGTAGTGAAGA-3′
	Reverse	5′-CCTGAAAGCCGATGTGGT-3′
Nrf2	Forward	5′-GTATGCAACAGGACATTGAGCAAG-3′
	Reverse	5′-TGGAACCATGGTAGTCTCAACCAG-3′
Sirt1	Forward	5′-TGTGGTAGAGCTTGCATTGATCTT-3′
	Reverse	5′-GGCCTGTTGCTCTCCTCATT-3′

### ALP staining

Following treatment, the cells were cultured in the osteogenic medium for 14 days and fixed with 4% formaldehyde at room temperature for 30 min and washed with 1 × PBS three times. The cells were stained with alkaline phosphatase (ALP, Beyotime Institute of Biotechnology, Shanghai, China) solution for 30 min at room temperature following the manufacturer's instructions. The photos were taken by an optical microscope (Olympus IX71, Tokyo, Japan).

### In-Cell Western analysis

Immediately after treatment, cells were washed in 1 × PBS and fixed in 10% neutral buffered formalin (NBF, Cellpath, Newtown, UK) in 1 × PBS for 20 min before being stained with the CellTag 700 staining ICW Kit I (Li-Cor Biosciences, Lincoln, NE). The samples were then incubated with the mouse anti-human Sirt1 antibody (1:600, Thermo Fisher, Waltham, MA) at 4 °C overnight with gentle shaking, followed by extensive washing in 1 × PBS containing 0.1% Tween 20 (Sigma-Aldrich, Shanghai, China) five times for 5 min per wash. The IRDye 800 CW goat anti-mouse secondary antibody (1:800) with the CellTag™ 700 stains (1:500) were added for 1 h at room temperature with gentle shaking followed by washing in 1 × PBS containing 0.1% Tween 20 for 5 min per wash. The plate was scanned on the Odyssey SA Imaging System (Li-Cor Biosciences, Lincoln, NE) using both 700 and 800 nm detection channels. Image Studio version 5 (Li-Cor Biosciences, Lincoln, NE) was used for performing the quantitative In-Cell Western (ICW) analysis.

### Immunofluorescence labelling of Sirt1

Cells (2.5 × 10^4^ cells/cm^2^) were seeded in eight-well multi-chambers (Falcon™ Culture Slides, Corning, Corning, NY) (*n* = 3) and cultured for 24 h. After treatment, the cells were fixed by 4% paraformaldehyde for 10 min at room temperature. The cells were washed three times with 1 × PBS and permeabilized by 0.2% Triton X-100 for 1 min. The cells were blocked with 10% goat serum (100 µL) (diluted in 1% BSA in 1 × PBS) (Dako, Nowy Sącz, Poland) for 30 min and washed five times with 1 × PBS before incubation in primary antibody (200 µL) (mouse anti-human Sirt1, 1:200 in 1% BSA) (Sigma-Aldrich, Shanghai, China) overnight at 4 °C. After rinsing with 1 × PBS, cells were incubated with Alexa-blue conjugated Goat Anti-Mouse secondary antibody (200 µL) (Alexa Fluor^®^ 488, Invitrogen, Carlsbad, CA, 1:50 in 1% BSA-PBS) for 1 h at room temperature in darkness. The slides were examined with an inverted fluorescent microscope (ZEISS, AX-10, Oberkochen, Germany).

### Animals and tissue sections

All animal studies were conducted following international standards on animal welfare and approved by the Animal Research Committee of Guangdong Medical University (Zhanjiang, China). The study was approved by the suggestion of animal research ethics (GDY20023l0) at Guangdong Medical University. Forty female Kunming mice (1-month-old and 12-months-old) were arranged and randomly assigned to four groups: Old (12-months-old), Old-RSV (12-months-old + RSV), Young (1-month-old) and Young-RSV (1-month-old + RSV). Cranium defects (1.0 mm) were created in the parietal bones of mice (*n* = 5 per group). Mice in the Old-RSV and Young-RSV groups were injected intraperitoneally with 0.2 mL RSV of 20 mg/kg/d for seven days, while the mice in the Old and Young groups were intraperitoneally injected with normal saline 0.2 mL. The mice were euthanized at 4 weeks after the surgery. The parietal bones were harvested and fixed for 24 h in 4% paraformaldehyde for further study.

### Micro-computed tomography

Micro-computed tomography (micro-CT) analysis was performed as described previously (Zhang et al. 2019). Briefly, the images were obtained via *ex vivo* micro-CT systems (Skyscanner 1174; Skyscan, Aartselaar, Belgium). Each sample was placed in a sample holder with the sagittal suture oriented parallel to the image plane and scanned in the air using the aluminium filer (0.25 mm), isotropic voxels (13 μm), 1000 ms integration time and one frame average. The scanner was equipped with an 80 kV, 500 μA X-ray tube, and a-36.9 megapixel Calibrate Centre offset coupled to a scintillator. For three-dimensional reconstruction (NRecon software, Skyscanner, Edinburgh, UK), the greyscale was set from 50 to 140. Standard three-dimensional morphometric parameters were determined in the ROI (100 cuts; 2.5 mm circle). Representative three-dimensional images were created using CT vox software (Skyscan, Edinburgh, UK).

### Nonlinear optical microscope observation

The excitation source of the SHG microscope was used to be pumped by a mode-locked Ti:sapphire laser oscillator (Spectra-Physics, 80 fs, 80 MHz and average powers up to 2.9 W). The laser beam was coupled into a multiphoton fluorescence scanning microscope (BX61 + FV1200, Olympus, Tokyo, Japan). The femtoseconds laser beam was focussed onto the sample by the objective lens (10× U PlanSApo, 0.40 N.A.; Olympus, Tokyo, Japan) with 10 mW. The SHG signal was isolated from the fundamental and any fluorescence by a band-pass filter (400/10 nm) and detected using a photomultiplier tube in the backscattered light path. Images were 800 × 800 pixels with 2 μs/pixel dwell time.

### Immunohistochemistry

The samples were dehydrated through a graded series of ethanol solutions before they were embedded in paraffin, which was cut parallel to the cross into five sections for Sirt1 immunohistochemistry. The slides were incubated with primary antibodies anti-Sirt1 (Abcam, Waltham, MA; 1:150). The two-step plus Poly-HRP Anti Rabbit/Mouse IgG Detection System (Elabscience, Houston, TX) was used to detect immunoreactivity. The slides were counterstained with Mayer's haematoxylin (Hongqiaolexiang Inc., Shanghai, China) and cover-slipped using a permanent mounting medium.

### Statistical analysis

The statistical analysis was carried out by a two-tailed pair Student's *t*-test using GraphPad Prism 8.0 (GraphPad Software, La Jolla, CA). *p* Value <0.05 was considered statistically significant.

## Results

### Sirt1 was decreased in oxidative stress

**Figure 1.**
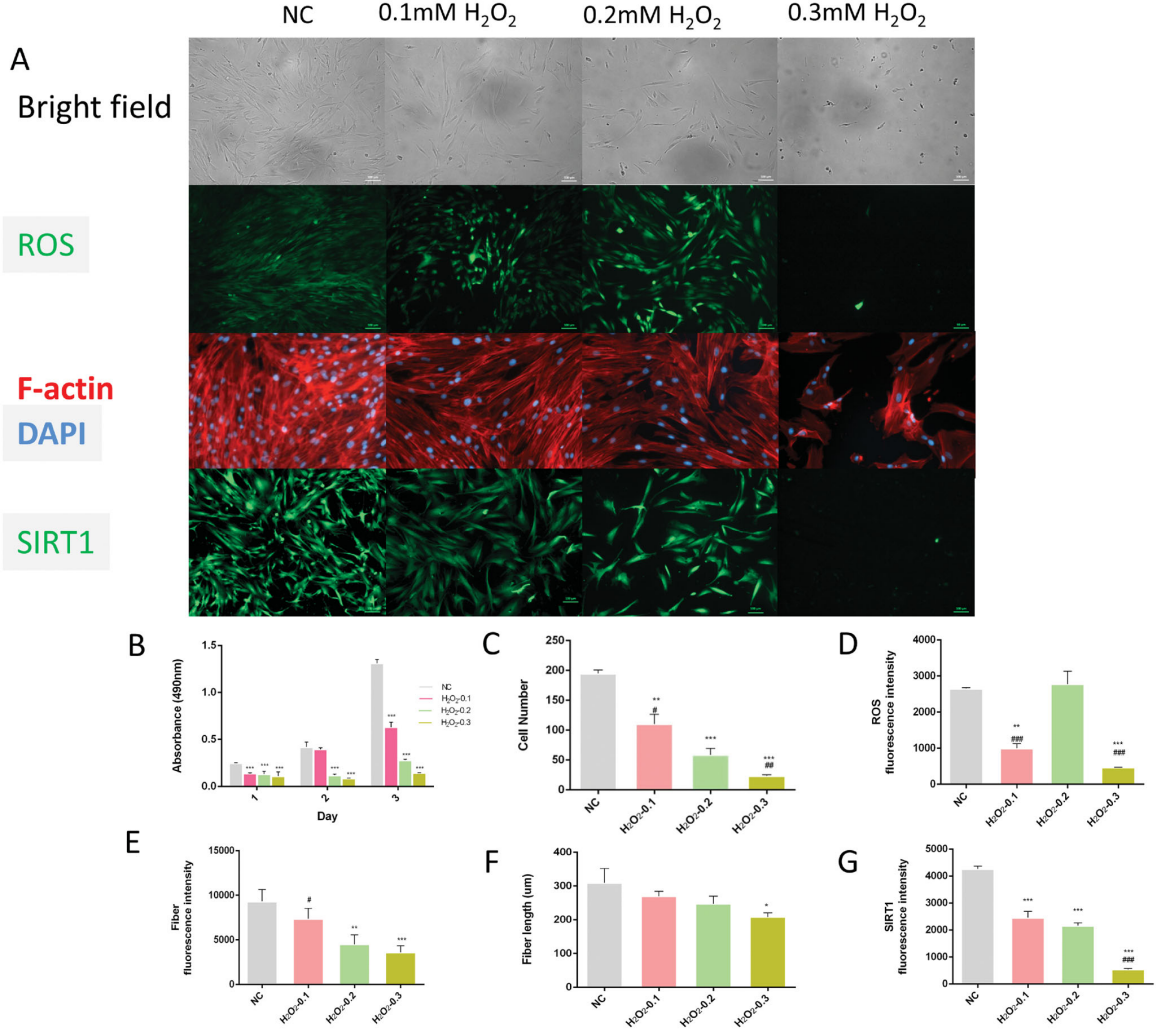
The effect of H_2_O_2_ on cell morphology, ROS production, F-actin and Sirt1 protein in hDPSCs. (A) The morphology of hDPSCs treated with H_2_O_2_ detected by immunofluorescence, cell panels: (1) under bright field, (2) ROS, (3) F-actin, (4) Sirt1 (scale bar: 100 μM); (B) cell proliferation of hDPSCs shown on day 1, 2 and 3 of treatment with H_2_O_2_; (C) MTT assay showing cell viability at different concentrations of H_2_O_2_ (0.1, 0.2 and 0.3 mM); (D) ROS fluorescence intensity at different concentrations of H_2_O_2_ (0.1, 0.2 and 0.3 mM); (E) fibre fluorescence intensity at different concentrations of H_2_O_2_ (0.1, 0.2 and 0.3 mM); (F) fibre length at different concentrations of H_2_O_2_ (0.1, 0.2 and 0.3 mM); (G) Sirt1 fluorescence intensity at different concentrations of H_2_O_2_ (0.1, 0.2 and 0.3 mM) (*p < 0.05, **p < 0.01, ***p < 0.001 vs. NC group; ^#^*p* < 0.05, ^##^*p* < 0.01, ^###^*p* < 0.001 vs. H_2_O_2_-0.2 mM group).

The concentration of H_2_O_2_ was optimized to induce oxidative stress, which was confirmed by the accumulation of ROS within the cells and cellular proliferation. After the treatment with H_2_O_2_ (0.2 mM), positive staining for ROS was located within the nuclei and cytoplasm of the cells (green). The ROS fluorescence intensity in the H_2_O_2_ (0.2 mM) group was stronger than the other groups (Figure 1(A,D)). Meanwhile, the fluorescence intensity of Sirt1 decreased in the H_2_O_2_ pre-treated group, which was localized within the nuclei and cytoplasm of the cells (Figure 1(A,G)).

### RSV decreased oxidative stress of hDPSCs

**Figure 2.**
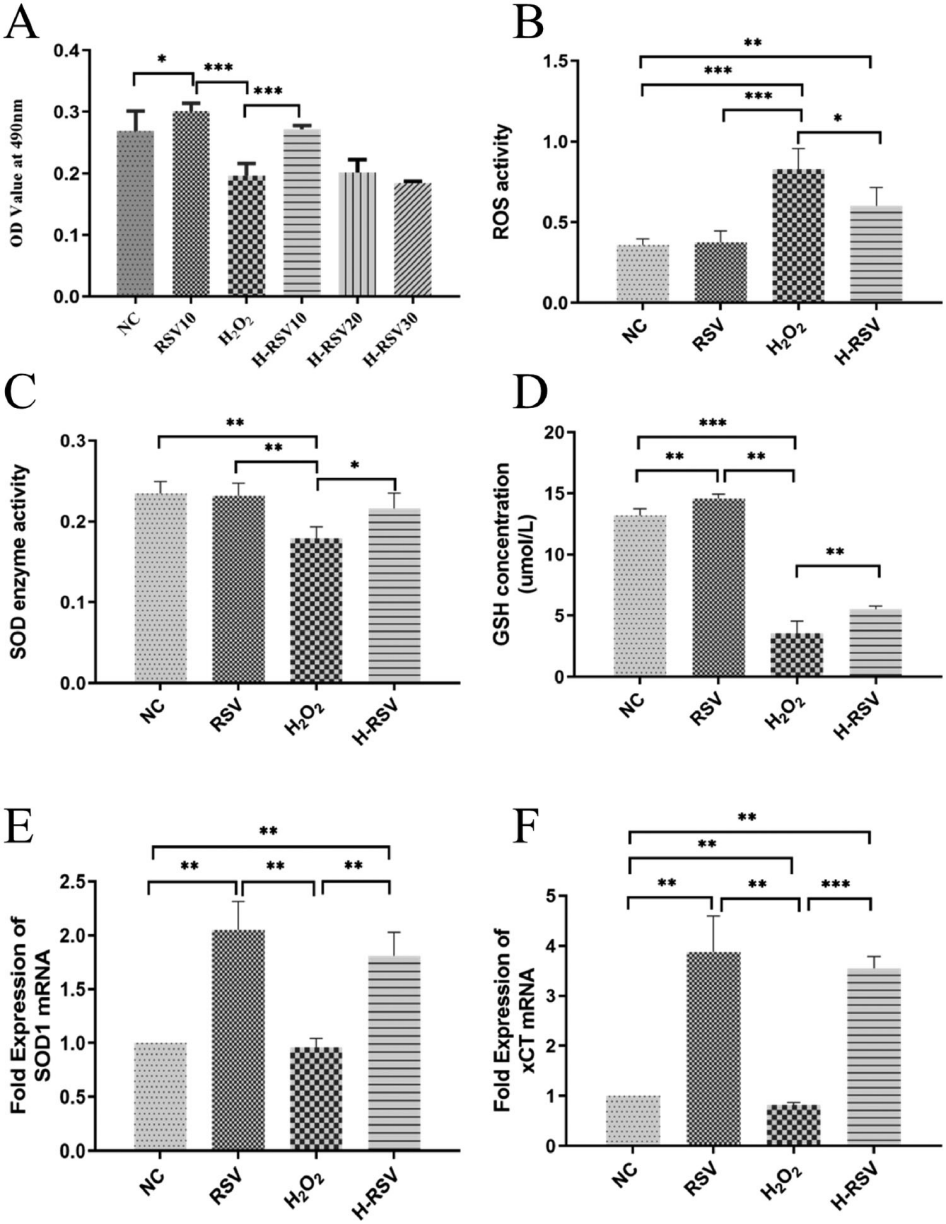
RSV promoted the proliferation and reduced the oxidative stress of hDPSCs pre-treated with H_2_O_2_. (A) Cell proliferation of hDPSCs pre-treated with/without H_2_O_2_ and different concentrations of RSV. (B) The ROS activity of hDPSCs pre-treated with/without H_2_O_2_ and RSV. (C) The SOD enzyme activity of hDPSCs pre-treated with/without H_2_O_2_ and RSV. (D) The GSH concentration of hDPSCs pre-treated with/without H_2_O_2_ and RSV. (E) The fold expression of SOD1 mRNA in hDPSCs. (F) The fold expression of xCT mRNA in hDPSCs. Group: NC (untreated cells), RSV (hDPSCs cultured with 10 μM RSV for 24 hours), H_2_O_2_ (hDPSCs treated by 0.2 mM H_2_O_2_ for 24 hours) and H-RSV (hDPSCs treated by 0.2 mM H_2_O_2_ cultured for 24 hours and then 10 μM RSV cultured for 24 hours) (*p < 0.05, **p < 0.01, ***p < 0.001 vs. NC group).

To investigate the effect of RSV against oxidative stress, the concentration of RSV was optimized by cell viability assay. The cell viability increased significantly in the RSV10 group than NC and H-RSV 10 than H_2_O_2_ group and the IC_50_ of RSV in hDPSCs is 67.65 ± 9.86. There were significant differences between the H_2_O_2_ group and H-RSV 10 group, and RSV (10 μM) was determined for the following experiments (Figure 2(A)). The ROS activity was declined (Figure 2(B)), the SOD enzyme activities were increased (Figure 2(C)) and the GSH concentration displayed enhancement (Figure 2(D)) in the H-RSV group compared with the H_2_O_2_ group. Similarly, further data showed that SOD1 and xCT mRNA expression increased significantly in the H-RSV group compared with the H_2_O_2_ group (Figure 2(E,F)).

### RSV promotes the expression of osteogenic genes

**Figure 3.**
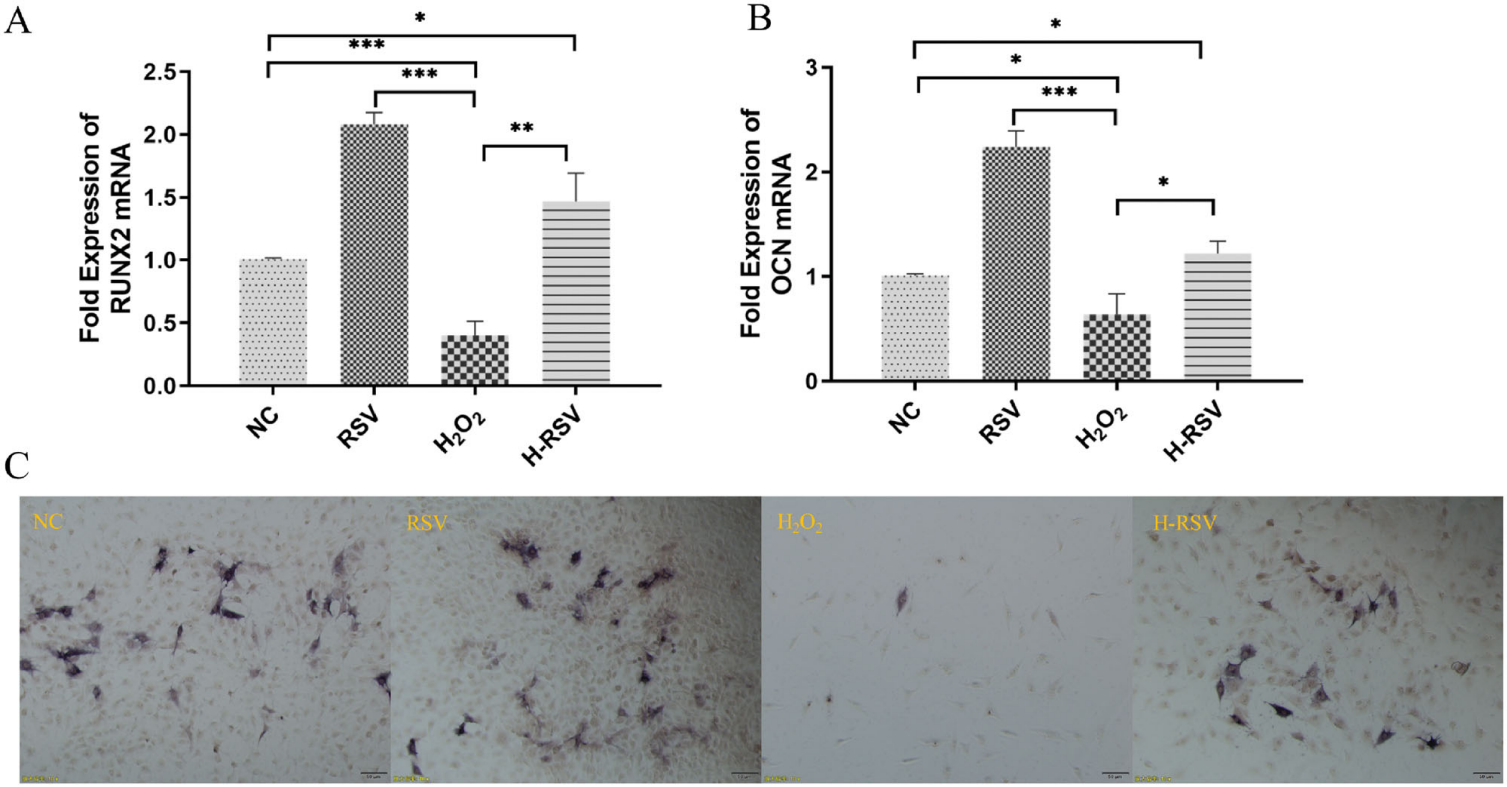
RSV promoted the osteogenic differentiation of hDPSCs after the treatment of H_2_O_2_. (A) The fold expression of Runx2 mRNA of hDPSCs pre-treated with/without H_2_O_2_ and RSV. (B) The fold expression of OCN mRNA of hDPSCs pre-treated with/without H_2_O_2_ and RSV. (C) The ALP staining of hDPSCs pre-treated with/without H_2_O_2_ and RSV (*p < 0.05, **p < 0.01, ***p < 0.001 vs. NC group).

**Figure 4.**
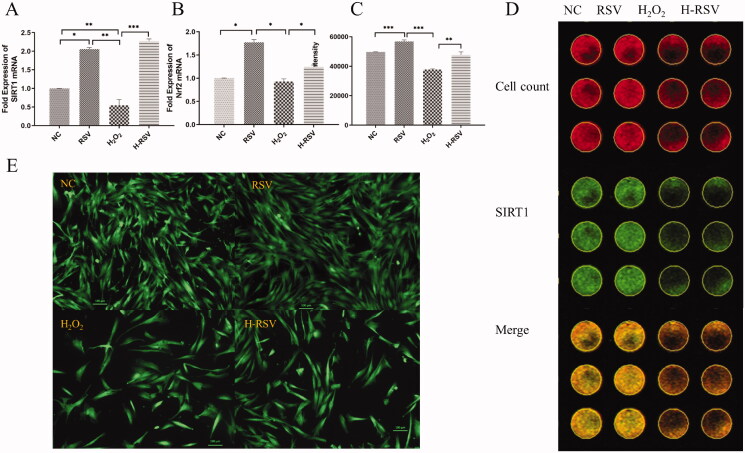
RSV increased the Sirt1 and Nrf2 mRNA expression in hDPSCs by pre-treated with H_2_O_2_. (A) The fold expression of Runx2 mRNA of hDPSCs pre-treated with/without H_2_O_2_ and RSV. (B) The fold expression of OCN mRNA of hDPSCs pre-treated with/without H_2_O_2_ and RSV. (C) Fluorescence intensity of In-Cell Western assay (ZEISS microscope AX-10). (D) The sirt1 fluorescence of hDPSCs pre-treated with/without H_2_O_2_ and RSV by In-cell western. (E) Sirt1 immunofluorescence staining of hDPSCs pre-treated with/without H_2_O_2_ and RSV (*p < 0.05, **p < 0.01, ***p < 0.001 vs. NC group).

The mRNA expression of Runx2, OCN, Sirt1 and Nrf2 increased significantly in the H-RSV group compared with the H_2_O_2_ group (Figures 3(A,B) and 4(A,B)). Moreover, The ALP staining was significantly stronger by RSV with/without pre-treated with H_2_O_2_ (Figure 3(C)). Immunofluorescent staining and ICW showed that Sirt1 fluorescence intensity increased significantly by RSV compared with/without pre-treated with H_2_O_2_ (*p* < 0.05) (Figure 4(C–E)).

### RSV promotes bone mass in mice

**Figure 5.**
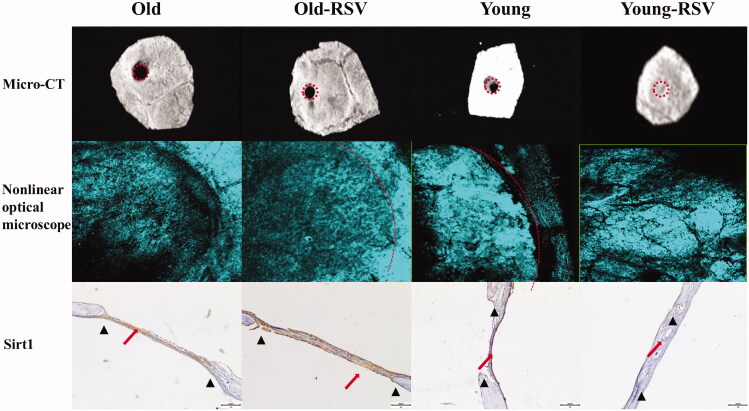
RSV promotes bone repair and increases bone mass. (A) Representative micro-CT images of bone repair and bone mass in calvarial defects. (B) Representative nonlinear optical microscope images of collagen in calvarial defects. (C) Immunohistochemical staining with sirt1 in hDPSCs pre-treated with/without H_2_O_2_ and RSV.

The area of the bone defect is significantly reduced in the 1-month-old + RSV group than the 1-month-old group, similarly in the 12-month-old + RSV group than the 12-month-old group. The intensity of collagen (green fluorescence) was stronger in the 12-month-old + RSV group than the 12-month-old group and stronger in the 1-month-old + RSV group than the 1-month-old group. The expression of Sirt1 was higher in the 12-month-old + RSV than the 12-month-old group (Figure 5).

## Discussion

Our present studies showed that hDPSCs pre-treated with H_2_O_2_ significantly reduced proliferation activity and increased oxidative stress. This finding was consistent with H_2_O_2_ induced oxidative stress in MC3T3-E1 cells (Choi et al. 2018). Previous studies also demonstrated that MSCs, when subjected to oxidative stress, significantly reduced proliferation activity (Mahmoudinia et al. 2019). The antioxidant activities of RSV were confirmed through the SOD enzyme activity and GSH concentration assay both to the normal cells and the oxidative-stressed cells, as well as the mRNA expressions of SOD1 and xCT, which represent the redox state of the cells.

Furthermore, the up-regulated mRNA expression of RUNX2 and OCN, and the stronger ALP staining in hDPSCs treated by RSV with or without H_2_O_2_ pre-treatment, suggest that the RSV promoted osteogenesis of hDPSCs. *In vivo*, the collagen and bone matrix of the mouse calvarial defect area was significantly increased after RSV treatment, indicating that RSV could promote bone formation in young and ageing mice. This result is consistent with the previous study about RSV preventing bone loss (Su et al. 2017).

Our results showed that the mRNA and protein expression of Sirt1 decreased in oxidative stress. However, these were increased by the treatment of RSV both *in vitro* and *in vivo*. Other studies also found that RSV could increase bone density via activating Sirt1 in mice (Liu et al. 2012). Besides, the Nrf2 mRNA expression was also enhanced by the treatment of RSV. Recent studies confirmed that Sirt1 regulates Nrf2 activation in the process of oxidative stress (Shah et al. 2017). Studies have confirmed that Nrf2 is a downstream target gene of Sirt1 (Wang et al. 2020). Taken together, these findings suggested that RSV could enhance osteogenesis via the Sirt1–Nrf2 pathway, indicating its therapeutic implication in anti-oxidative stress or other bone-related diseases.

## Conclusions

These findings shed light on the potential applications of RSV in stem cells-based therapy, with a higher proliferation rate and greater osteogenic potential of hDPSCs in regenerative medicine. Moreover, RSV might have the effect of promoting bone formation for the elderly or patients with oxidative stress physiological states such as hypertension, heart disease, diabetes, etc., as a potential agent.

## Data Availability

The datasets used or analysed during the current study are available from the corresponding author on reasonable request.
